# A dataset of winter wheat aboveground biomass in China during 2007–2015 based on data assimilation

**DOI:** 10.1038/s41597-022-01305-6

**Published:** 2022-05-11

**Authors:** Hai Huang, Jianxi Huang, Xuecao Li, Wen Zhuo, Yantong Wu, Quandi Niu, Wei Su, Wenping Yuan

**Affiliations:** 1 College of Land Science and Technology, China Agricultural University, Beijing, 100083 China; 2 Key Laboratory of Remote Sensing for Agri-Hazards, Ministry of Agriculture and Rural Affairs, Beijing, 100083 China; 3 State Key Laboratory of Sever Weather, Chinese Academy of Meteorological Sciences, Beijing, 100081 China; 4 School of Resources and Environment, University of Electronic Science and Technology of China, Chengdu, 611731 China; 5 School of Atmospheric Sciences, Sun Yat-sen University, Guangzhou, 510245 Guangdong China

**Keywords:** Agroecology, Biogeography

## Abstract

As a key variable to characterize the process of crop growth, the aboveground biomass (AGB) plays an important role in crop management and production. Process-based models and remote sensing are two important scientific methods for crop AGB estimation. In this study, we combined observations from agricultural meteorological stations and county-level yield statistics to calibrate a process-based crop growth model for winter wheat. After that, we assimilated a reprocessed temporal-spatial filtered MODIS Leaf Area Index product into the model to derive the 1 km daily AGB dataset of the main winter wheat producing areas in China from 2007 to 2015. The validation using ground measurements also suggests the derived AGB dataset agrees well with the filed observations, i.e., the R^2^ is above 0.9, and the root mean square error (RMSE) reaches 1,377 kg·ha^−1^. Compared to county-level statistics during 2007–2015, the ranges of R^2^, RMSE, and mean absolute percentage error (MAPE) are 0.73~0.89, 953~1,503 kg·ha^−1^, and 8%~12%, respectively. We believe our dataset can be helpful for relevant studies on regional agricultural production management and yield estimation.

## Abstract (summary)

**Table:** 

Measurement(s)	aboveground biomass
Technology Type(s)	data assimilation

## Background & Summary

Wheat is the most important food diet component for people in most countries. Winter wheat is a main variety of wheat. Monitoring the growth and production potential of winter wheat is important for global food security^1^. China is one of the world’s largest winter wheat producers, accounting for 85% of its total summer crop harvest. Stable and increasing winter wheat production in China is crucial to the sustainable development of global agriculture^2,3^. The aboveground biomass (AGB) is one of the most frequently used variables in monitoring agricultural ecosystems^4^. It is closely related to crop growth and yield formation and is an essential part of global climate change, carbon cycle, material flow, and energy exchange research. Accurately acquiring the temporal and spatial variability of AGB of winter wheat provides fundamental and important information for yield estimation and food security.

AGB is the total dead and living plant organs measured in units of dry mass per unit ground area. It is closely related to the net primary production (NPP), which is a widely used variable in ecosystem research and describes the ability of vegetation photosynthesis and carbon sequestration. AGB can be converted to total biomass (i.e., above and underground biomass) with a root-shoot ratio^5^, and NPP can be estimated by multiplying an empirical coefficient of 0.45^5^. Since most NPP products rarely distinguish specific crop types and have huge uncertainties^6^, AGB data can be helpful for NPP estimation and validation.

Estimation of crop AGB in previous studies can be mainly divided into two categories: based on crop growth models and remotely sensed observations. Many studies demonstrate the sufficient accuracy of crop growth models in crop AGB simulation at site scale^7–10^. The crop growth model shows more advantages for simulating AGB than other modeled crop variables such as leaf area index (LAI) and yield^11,12^. With improved gridded meteorological data, crop growth models can be extended to a large regional scale after calibration^13^. Although crop growth models can simulate the time-series data of AGB, their accuracies depend on reliable model calibration, which is usually difficult for large areas^14^. In addition, the spatial variability of the model results after calibration is only determined by the coarse-resolution weather-driven data, which further limits the improvement of spatial accuracy. However, remotely sensed observations can acquire a wide range of information and play an increasingly important role in crop AGB estimation^15^. Multiple studies show that the crop AGB can be estimated using the strong correlations between measured AGB and remote sensing vegetation indices at various spatial scales^16–21^. Besides, the developments of physical models make it possible to describe the relationship between crop and remote sensing response without existing training data, especially for radar remote sensing^22^. Regional AGB estimation studies based on remote sensing often have finer spatial resolution than crop growth models. However, these methods based on correlations between AGB and remote sensing variables usually rely on many field samples and have poor temporal and spatial generalization capabilities.

Data assimilation (DA) techniques provide a well-understood way to combine the advantage of process-based crop growth models with remote sensing data^14,23,24^. Many studies have been conducted to improve the simulated results from crop growth models for both the spatial details and the overall accuracy by assimilating the satellite-derived LAI at a regional scale^3,25–27^. Previous studies have also proved the effectiveness of assimilating remote sensing observations into crop growth models to better simulate the regional crop AGB^28–31^.

**Fig. 1:**
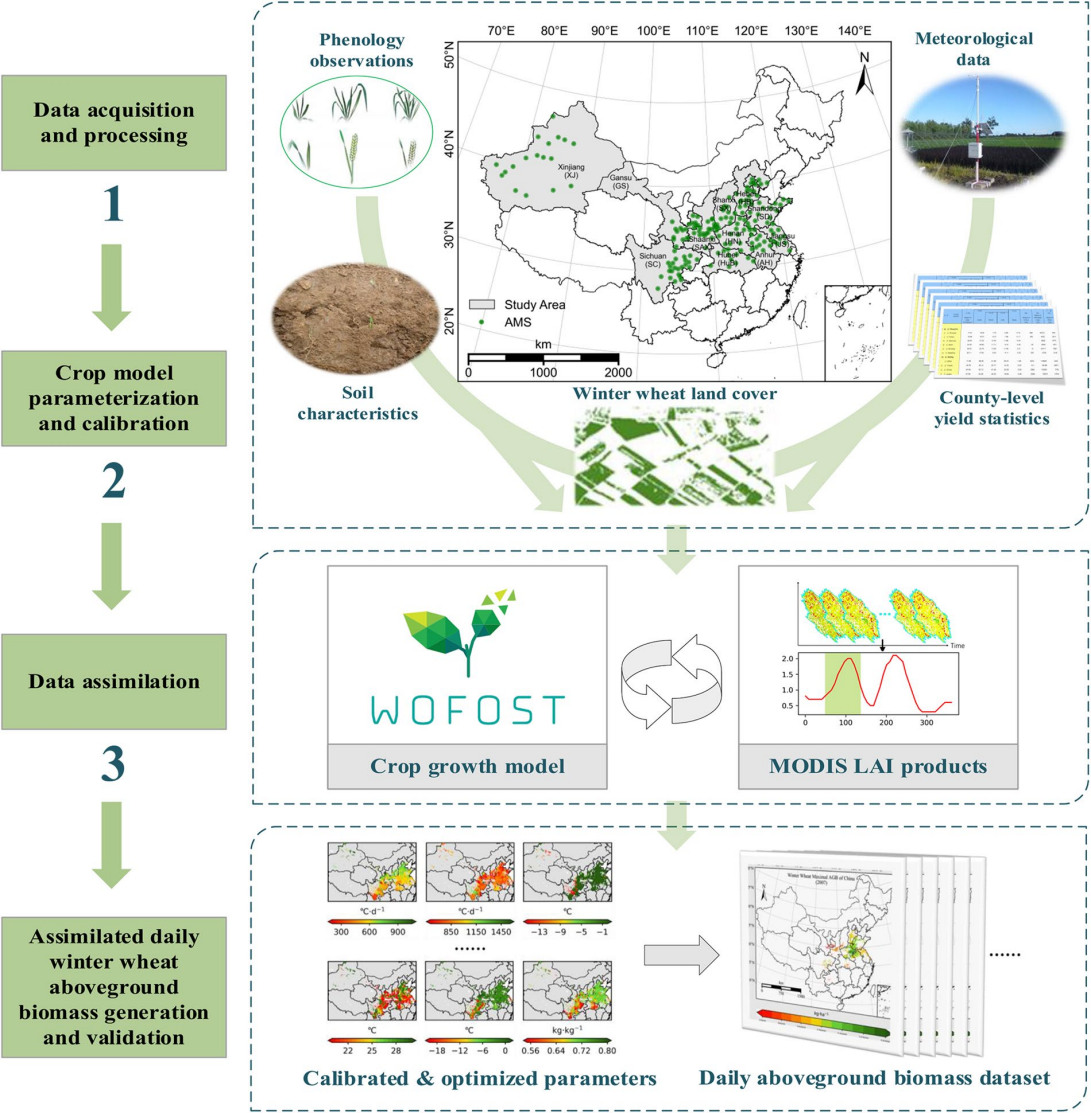
Schematic diagram outlining the inputs, major processing steps used, and generated outputs.

In this research, we firstly calibrated the World Food Studies simulation model (WOFOST) across different zones based on many site observations from agricultural meteorological station networks and the county-level statistical data. Then, we assimilated a reprocessed temporal-spatial filtered MODIS LAI product^32^ into the calibrated WOFOST model by recalibrating three parameters related to phenology and leaf dynamics at 1 km resolution. Finally, we generated the daily AGB dynamic data of winter wheat at 1 km resolution during 2007–2015 (Fig. 1). This dataset is the first large-region winter wheat AGB product generated by assimilating remote sensing data and a crop growth model. The product can deepen the understanding of winter wheat production dynamics and variability and the agricultural system modeling over a long period and in a large area.

## Methods

We selected eleven major wheat production provinces of China for the study area, which comprise the largest winter wheat-sowing fraction: Henan, Shandong, Anhui, Jiangsu, Hebei, Hubei, Shanxi, Shaanxi, Sichuan, Xinjiang, and Gansu (Fig. 1). The wheat planting area is about 22 million ha in these provinces, accounting for more than 93% of the total wheat planting area. The total wheat production in these regions contributes more than 96% of the total wheat production in China, with more than 128 million tons in 2019^33^.

We developed a methodological framework for high-resolution AGB mapping. It mainly includes three parts: (1) Data acquisition and processing. (2) The WOFOST model parameterization and calibration. (3) Data assimilation (Fig. 1). Each part is explained in more detail below.

### Data acquisition and processing

#### Meteorological data

China Meteorological Forcing Dataset^34,35^ is used as weather driving data for the WOFOST model. The dataset is based on the internationally existing Princeton reanalysis data, Global Land Data Assimilation System data, Global Energy and Water Cycle Experiment-Surface Radiation Budget radiation data, and Tropical Rainfall Measuring Mission precipitation data. It is made by fusing the conventional meteorological observation data of the China Meteorological Administration. It includes seven elements: near-surface air temperature, air pressure, near-surface total humidity, wind speed, ground downward shortwave radiation, ground downward longwave radiation, and ground precipitation rate. The meteorological drive elements required for WOFOST are daily radiation, minimum temperature, maximum temperature, water vapor pressure, average wind speed, and precipitation. Details of these variables that participated in the WOFOST model can be referred to in Table S1.

#### Soil characteristics measurements and phenology observations

Soil and phenology data were collected at 177 agricultural meteorological stations (AMS) from 2007 to 2015 (Fig. 1). Soil characteristics include soil moisture content at wilting points, field capacity, and saturation. To be consistent with the corresponding units in the crop model, the original data in weight water content was converted into volume water content through the corresponding soil bulk density measurements. Winter wheat phenology observations include the date of emergence (more than 50% of the wheat seedlings in the field show the first green leaves and reached about 2 cm), anthesis (the inner and outer glumes of the middle and upper florets of more than 50% of the wheat ears in the whole field are open, and the anthers loose powder), and maturity (more than 80% of the wheat grains turn yellow, the glumes and stems turn yellow, and only the upper first and second nodes are still slightly green). In most cases, the phenological stage “anthesis” is missing. The anthesis date was calculated by adding seven days to the observed heading date (when more than 50% of the wheat in the whole field exposes the tip of the ear from the sheath of the flag leaf).

#### County-level yield statistics data

The county-level yield data was collected from city statistical yearbooks of the study area from 2007 to 2015. Since most statistical yearbooks do not directly record per-unit yield data, the county-level yield was obtained by dividing the total yield and planting area. It is worth noting that all yields were calculated in units of metric kilograms per cultivated hectares (kg·ha^−1^).

#### The winter wheat land cover data

We used a winter wheat land cover product from a 1 km resolution dataset named ChinaCropArea1km^36^. This data was derived from GLASS leaf area index products and crop phenology from 2000 to 2015. This dataset is the base map of our data production.

#### The MODIS LAI data

We used the improved 8-days MODIS LAI products (i.e., 1 km) generated by Yuan *et al*.^32^ to assimilate the WOFOST model. The products applied the modified temporal-spatial filter and Savitzky-Golay filter to overcome the spatial-temporal discontinuity and inconsistence of raw MODIS LAI products, which makes them more applicable for the realm of land surface and climate modeling. The products can be accessed via the Land-Atmosphere Interaction Research Group website at Sun Yat-sen University (http://globalchange.bnu.edu.cn/research/lai).

### The WOFOST model parameterization and calibration

#### The WOFOST model introduction

**Fig. 2:**
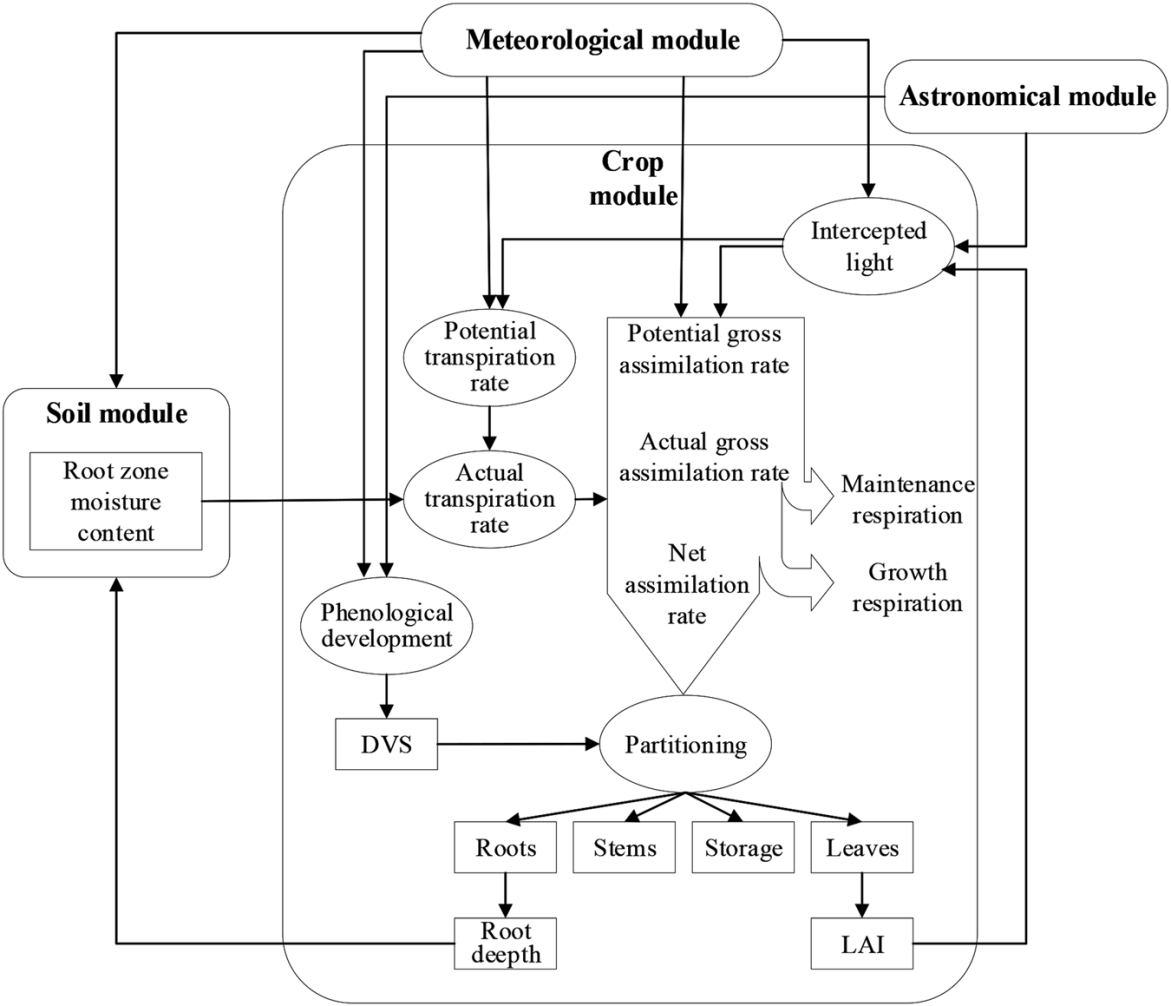
Schematic overview of the major processes implemented in WOFOST. The Astronomical module calculates day length, some variables relating to solar elevation, and the fraction of diffuse radiation.

The WOFOST model was initially developed as a crop growth simulation model to evaluate the yield potential of various crops in tropical countries^37^. In this study, we chose the WOFOST model because the model reaches a trade-off of the complexity of the crop model and is suitable for large-scale simulations^3^. The WOFOST model is a typical crop growth model that explains crop growth based on underlying processes such as photosynthesis and respiration and their response to changing environmental conditions^38^. The WOFOST model estimates phenology, LAI, aboveground biomass, and storage organ biomass (i.e., grain yield) at a daily time step^39^ (Fig. 2).

#### Zonal parameterization

We first divided the study area covered by AMS into seamless Thiessen polygon zones. Each Thiessen polygon contains only a single AMS. These zones represent the whole areas where any location is closer to its associated AMS point than any other AMS point. Then, we assigned parameters to the entire zone based on the AMS data, including crop calendar (date of emergence) and soil water retention parameters (soil moisture content at wilting point, field capacity, and saturation). Besides, we also optimized two main crop parameters for controlling phenological development stages, namely TSUM1 (accumulated temperature required from emergence to anthesis) and TSUM2 (accumulated temperature required from anthesis to maturity), by minimizing the cost function of the observational and simulated date corresponding to anthesis and maturity.

#### Parameter calibration within a single zone

**Fig. 3:**
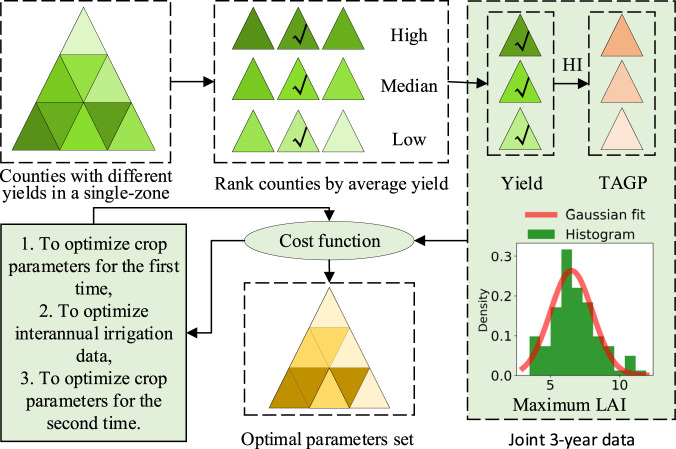
Flow chart of parameter calibration within a single zone.

We implemented the calibration of parameters within every single zone, as illustrated in Fig. 3. We calculated the average statistical yield of each county within every single zone from 2007 to 2015, then ranked the counties in descending order and divided them into three groups, namely high, medium, and low-level yield counties, by the 33% quantile and 67% quantile of the average statistical yield. The three counties corresponding to 17% quantile, 50% quantile, and 83% quantile would be used for subsequent calibration and represent the corresponding three yield level groups. We used the statistical yields (converted to dry matter mass based on the standard moisture content of 12.5%) of the three counties for multiple years and a harvest index for each province to convert the county-level yield to AGB for calibration. The harvest index of each province was mainly estimated from the AMS’s dynamic growth records on the biomass composition of the dominant winter wheat varieties of the province and a published literature^40^. Besides, we collected the maximum LAI observations on all agrometeorological stations in all years in the study area, according to its histogram. We found that the histogram follows a normal distribution with a mean of 6.5 and a standard deviation of 1.5. Finally, we calibrated three sets of parameters corresponding to three yield level groups in each single zone according to the three selected counties.

We designed a three-step calibration strategy for a specific yield level group. Firstly, as winter wheat varieties did not change significantly according to information recorded by agrometeorological stations from 2007 to 2015, we assumed the crop parameters of winter wheat remain unchanged every three years to combine three years of observational data to calibrate the parameters of the WOFOST model better. We maximized a log-likelihood function based on the maximum LAI statistics and every three-year county-level yield and AGB data mentioned to optimize selected crop parameters (see Table S2 in the Supplement Materials).

The log-likelihood function was constructed as follows:

1 $$log\;{{\rm{L}}}_{{\rm{LAI}}}=-\frac{1}{2}\left[dlog\left(2\pi \right)+log\left(\left|{\Sigma }_{{\rm{LAI}}}\right|\right)+{\rm{MD}}{\left({{\bf{x}}}_{{\rm{LAI}}};{\mu }_{{\rm{LAI}}},{\Sigma }_{{\rm{LAI}}}\right)}^{2}\right]$$

2 $$log\;{{\rm{L}}}_{{\rm{TWSO}}}=-\frac{1}{2}\left[dlog(2\pi )+log\left(\left|{{\boldsymbol{\Sigma }}}_{{\rm{TWSO}}}\right|\right)+{\rm{MD}}{\left({{\bf{x}}}_{{\rm{TWSO}}};{{\boldsymbol{\mu }}}_{{\rm{TWSO}}},{{\boldsymbol{\Sigma }}}_{{\rm{TWSO}}}\right)}^{2}\right]$$

3 $$log\;{{\rm{L}}}_{{\rm{AGB}}}=-\frac{1}{2}\left[dlog(2\pi )+log\left(\left|{{\boldsymbol{\Sigma }}}_{{\rm{AGB}}}\right|\right)+{\rm{MD}}{\left({{\bf{x}}}_{{\rm{AGB}}};{{\boldsymbol{\mu }}}_{{\rm{AGB}}},{{\boldsymbol{\Sigma }}}_{{\rm{AGB}}}\right)}^{2}\right]$$

4 $$log\;{\rm{L}}=log\;{L}_{{\rm{LAI}}}+log\;{L}_{{\rm{TWSO}}}+log\;{L}_{{\rm{AGB}}}$$

Where *log* L is the natural logarithm of the likelihood function, d is the dimension, that is, the number of years of joint calibration, which is set to 3 in this study **x**_LAI_ is the vector composed of the maximum value of the 3-year LAI simulated by WOFOST, ***μ***_LAI_ and **Σ**_LAI_ are the mean vector and error covariance matrix of maximum LAI based on observation statistics. The annual maximum LAI was assumed to be independent, and the mean and standard deviation for each year was set the same as the result of Fig. 3. Similarly, **x**_TWSO_ and **x**_AGB_ are the yield vector and AGB vector at maturity of 3 years simulated by WOFOST, and ***μ***_TWSO_, ***μ***_AGB_ are their corresponding county-level statistic vector, **Σ**_TWSO_ and **Σ**_AGB_ are their corresponding error covariance matrix. In this study, we assumed that the annual yield or AGB was independent, and their corresponding standard deviation was 10% of their statistical value. |**Σ**| is the determinant of **Σ**. The expression ${\rm{MD}}{({\bf{x}};{\boldsymbol{\mu }},{\boldsymbol{\Sigma }})}^{2}={({\bf{x}}-{\boldsymbol{\mu }})}^{{\rm{T}}}{{\boldsymbol{\Sigma }}}^{-1}({\bf{x}}-{\boldsymbol{\mu }})$, where MD is the Mahalanobis distance between the point **x** and the mean vector ***μ***.

Secondly, we optimized the inter-annual irrigation. We optimized two parameters every year: the critical value of soil moisture (denoted as SMc) and the amount of irrigation (denoted as V). When the soil moisture simulated by WOFOST is lower than SMc, an irrigation event will be triggered, and the irrigation amount is V cm. In this study, we combined three-year data for calibration with six parameters for optimization. The optimization strategy is the same as the previous step by maximizing the log-likelihood function. Finally, we fixed the optimized irrigation parameters and repeated the first step to calibrate the selected crop parameters and obtain the final optimal parameters.

### Data assimilation

Considering that MODIS LAI is relatively low compared to the actual LAI of winter wheat^41^, we select a weak-constraint cost function based on the least square of normalized observational and simulated LAI as shown in Eq. (5), which is assimilating the trend information of MODIS LAI into the crop growth model.

5 $$J={\sum }_{{\rm{t}}=1}^{{\rm{n}}}{\left(\frac{{{\rm{LAI}}}_{{\rm{MODIS}}}^{{\rm{t}}}-{{\rm{LAI}}}_{{\rm{MODIS}}}^{min}}{{{\rm{LAI}}}_{{\rm{MODIS}}}^{max}-{{\rm{LAI}}}_{{\rm{MODIS}}}^{min}}-\frac{{{\rm{LAI}}}_{{\rm{WOFOS}}}^{{\rm{t}}}-{{\rm{LAI}}}_{{\rm{WOFOS}}}^{min}}{{{\rm{LAI}}}_{{\rm{WOFOS}}}^{max}-{{\rm{LAI}}}_{{\rm{WOFOS}}}^{min}}\right)}^{2}$$

Where ${{\rm{LAI}}}_{{\rm{MODIS}}}^{{\rm{t}}}$ and .. are MODIS LAI and WOFOST simulated LAI of time t. ${{\rm{LAI}}}_{{\rm{MODIS}}}^{max}$ and ${{\rm{LAI}}}_{{\rm{WOFOS}}}^{max}$ are maximum of MODIS LAI and WOFOST simulated LAI. ${{\rm{LAI}}}_{{\rm{MODIS}}}^{min}$ and ${{\rm{LAI}}}_{{\rm{WOFOS}}}^{min}$ are minimum of MODIS LAI and WOFOST simulated LAI. *J* is the value of the cost function.

We reinitialize the day of emergence (IDEM), the life span of leaves growing at 35 °C (SPAN), and thermal time from emergence to anthesis (TSUM1) in the WOFOST model on each 1 km winter wheat pixel according to cost function between WOFOST LAI and MODIS LAI. Besides, we applied the Subplex algorithm from the NLOPT library (https://github.com/stevengj/nlopt) for parameter optimization.

## Data Records

The generated winter wheat daily AGB dataset during 2007–2015 is available at 10.6084/m9.figshare.16680784.v3^42^. An overview of the data files showing the coverage, resolution, formats are shown in Table 1. The dataset is stored in GeoTiff format with the unit of kg·ha^−1^. Each GeoTiff file has 270 bands corresponding to the last 90 days of the previous year and the first 180 days of the current year.

**Table 1:** Data record information for gridded winter wheat daily aboveground biomass products.

Filename	Format	Resolution	Span
CHN_Winter_Wheat_AGB_2007.tif	GeoTiff	Daily, 0.01° × 0.01°	2006-10-03 - 2007-06-29
CHN_Winter_Wheat_AGB_2008.tif			2007-10-03 - 2008-06-28
CHN_Winter_Wheat_AGB_2009.tif			2008-10-03 - 2009-06-29
CHN_Winter_Wheat_AGB_2010.tif			2009-10-03 - 2010-06-29
CHN_Winter_Wheat_AGB_2011.tif			2010-10-03 - 2011-06-29
CHN_Winter_Wheat_AGB_2012.tif			2011-10-03 - 2012-06-28
CHN_Winter_Wheat_AGB_2013.tif			2012-10-03 - 2013-06-29
CHN_Winter_Wheat_AGB_2014.tif			2013-10-03 - 2014-06-29
CHN_Winter_Wheat_AGB_2015.tif			2014-10-03 - 2015-06-29

## Technical Validation

**Fig. 4:**
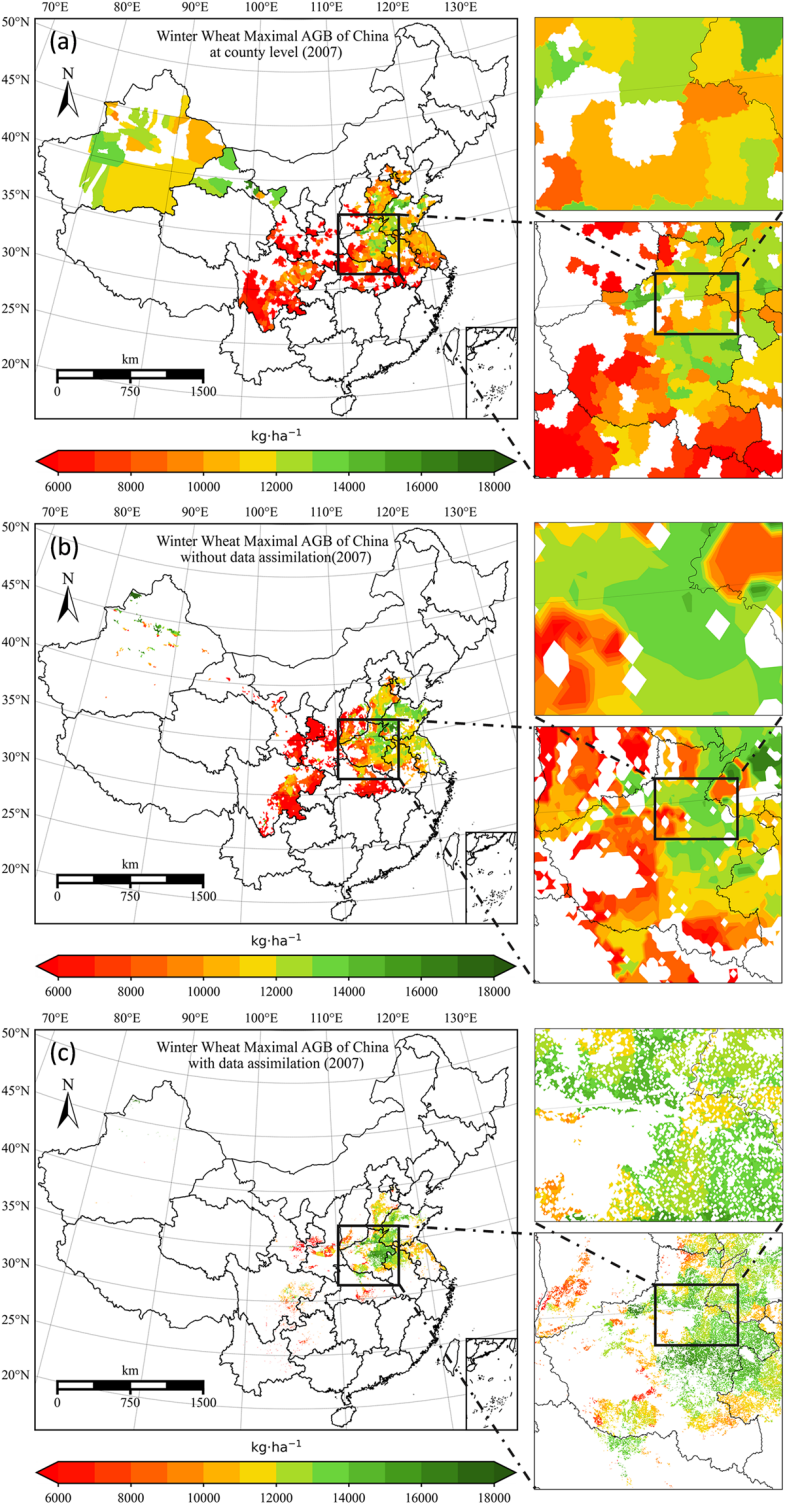
Map of total aboveground biomass of 2007. (**a**) the county-level result, it is generated by dividing the county-level statistical yield by the corresponding provincial-level harvest index, note that there are missing data for some counties; (**b**)result before data assimilation, it is generated by running WOFOST at 0.1° × 0.1° meteorological grid with calibrated parameters; (**c**) result after data assimilation, it is generated by assimilating MODIS LAI into WOFOST at 0.01° × 0.01° grid.

The assimilated result increases the spatial variability within the meteorological grid while preserving the spatial trend, as shown in Fig. 4. Before remote sensing data is assimilated into crop growth models, the main factor driving spatial variability is meteorological data. Compared with the county statistical results, the result before data assimilation has been able to grasp the overall spatial trend, but the expression ability of spatial details is relatively poor due to the coarse resolution of meteorological data.

The planting density of winter wheat in the northwest region (mainly in Xinjiang) is very sparse. The planting area only accounts for less than 0.5% of the total administrative area, so it looks like pixel information is lost on a 1km resolution map. In addition, since the model calibration is mainly carried out based on the statistical data of every three years, before data assimilation, some regions have obvious different spatial patterns compared with county-level statistics of the corresponding year. However, this difference is reduced after data assimilation as shown in the subgraph on the top right of Fig. 4(a,b),(c).

### AGB data validation using *in-situ* measurements

**Fig. 5:**
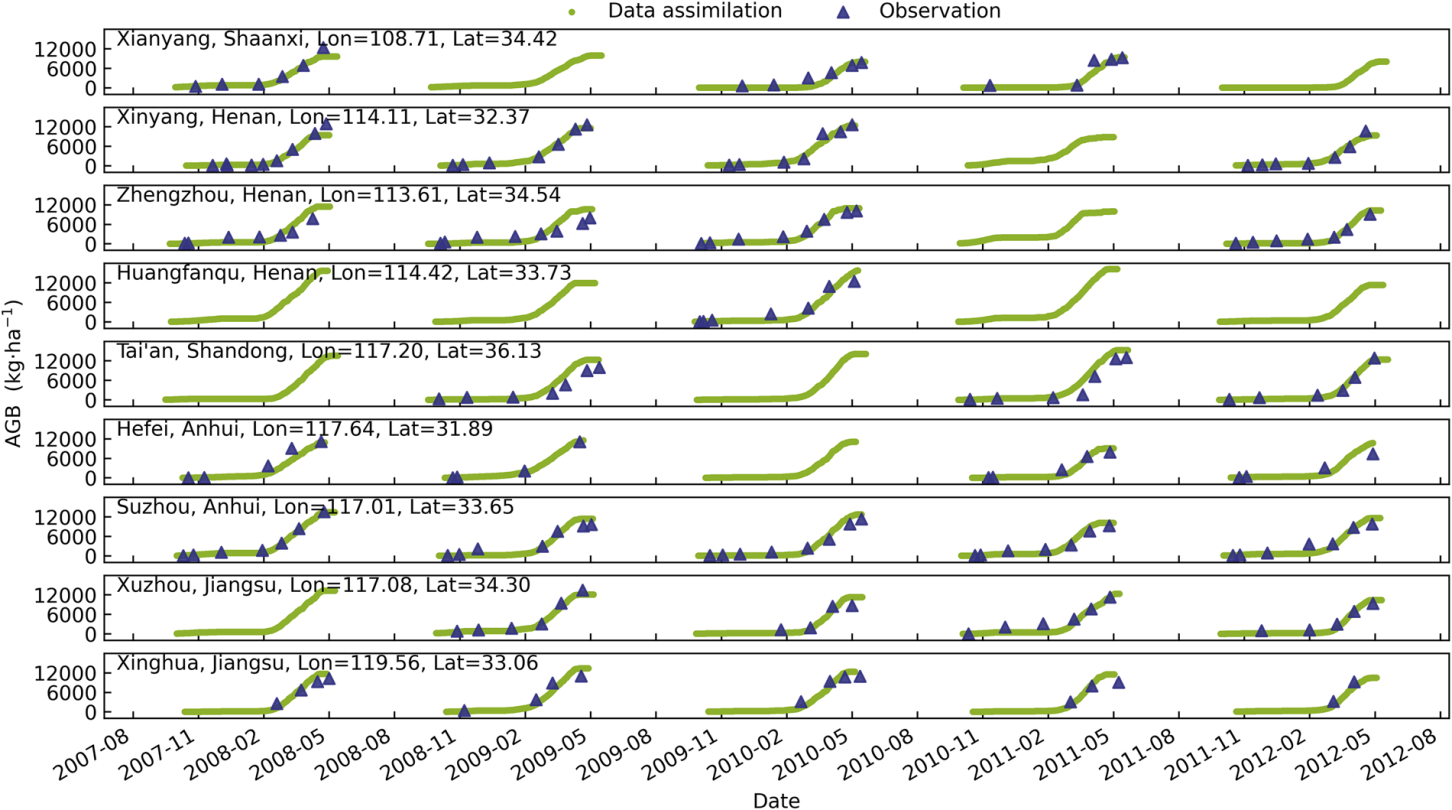
Time-series plot of simulated and observed aboveground biomass at nine agricultural meteorological stations from 2008 to 2012.

**Fig. 6:**
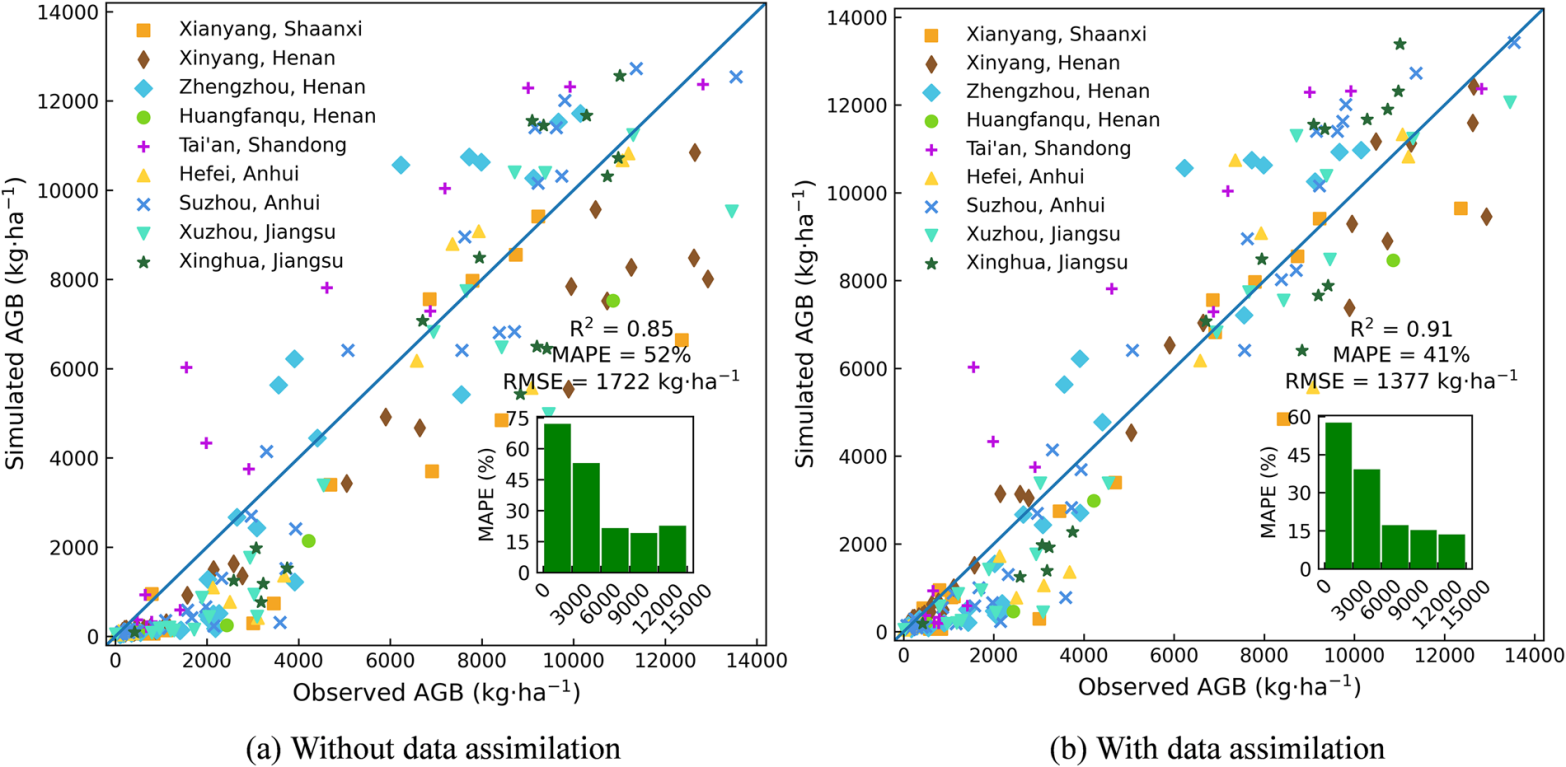
Comparison of the simulated aboveground biomass without and with data assimilation with observation at nine agricultural meteorological stations.

The generated AGB dataset is in high agreement with the station observed AGB (Fig. 5). The generated time-series result well captures the temporal characteristics of winter wheat AGB, especially the stage of a rapid increase in biomass accumulation. However, there are some overestimations or underestimations compared with some year- station’s measurements. For example, the AGB of Tai’an station in Shandong is consistently overestimated for most growth periods. The main reason is the scale difference between county statistics and station measurement data used for model calibration. Figure 6 shows the scatter plot of all nine stations’ observed and simulated values before and after data assimilation with the coefficient of determination (R^2^) from 0.85 to 0.91 and mean absolute percentage error (MAPE) from 52% to 41%, root mean square error (RMSE) from 1722 kg·ha^−1^ to 1377 kg·ha^−1^. The overall MAPE is relatively high, however, the MAPE varies considerably at different periods of the winter wheat growing season. The green bar subplots in Fig. 6 represent MAPE at different stages graded by AGB production every 3000 kg·ha^−1^ from zero to 15000 kg·ha^−1^. The results show that the MAPE decreases at all stages after data assimilation, and the MAPE of simulated AGB greater than 6000 kg·ha^−1^ decreased from an average of around 20% to around 15%. However, the simulated AGB is generally low, and the MAPE is large in the early part of the growing season (less than 6000 kg·ha^−1^). This is mainly because AGB in the early growth stage is especially sensitive to the parameter TDWI (initial total crop dry weight). However, there is large uncertainty in calibrating TDWI without observations in the early growth stage. Considering the scale effect between the remote sensing data and ground stations, this accuracy is encouraging to a certain extent. Detailed information on the AGB measurements is shown in Appendix S1 in the Supplement.

### AGB data validation using county-level statistics

**Fig. 7:**
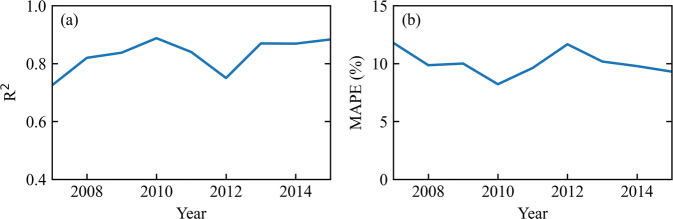
Accuracy of county-level validation for the maximum AGB from 2007 to 2015. (**a**) R^2^. (**b**) MAPE.

**Fig. 8:**
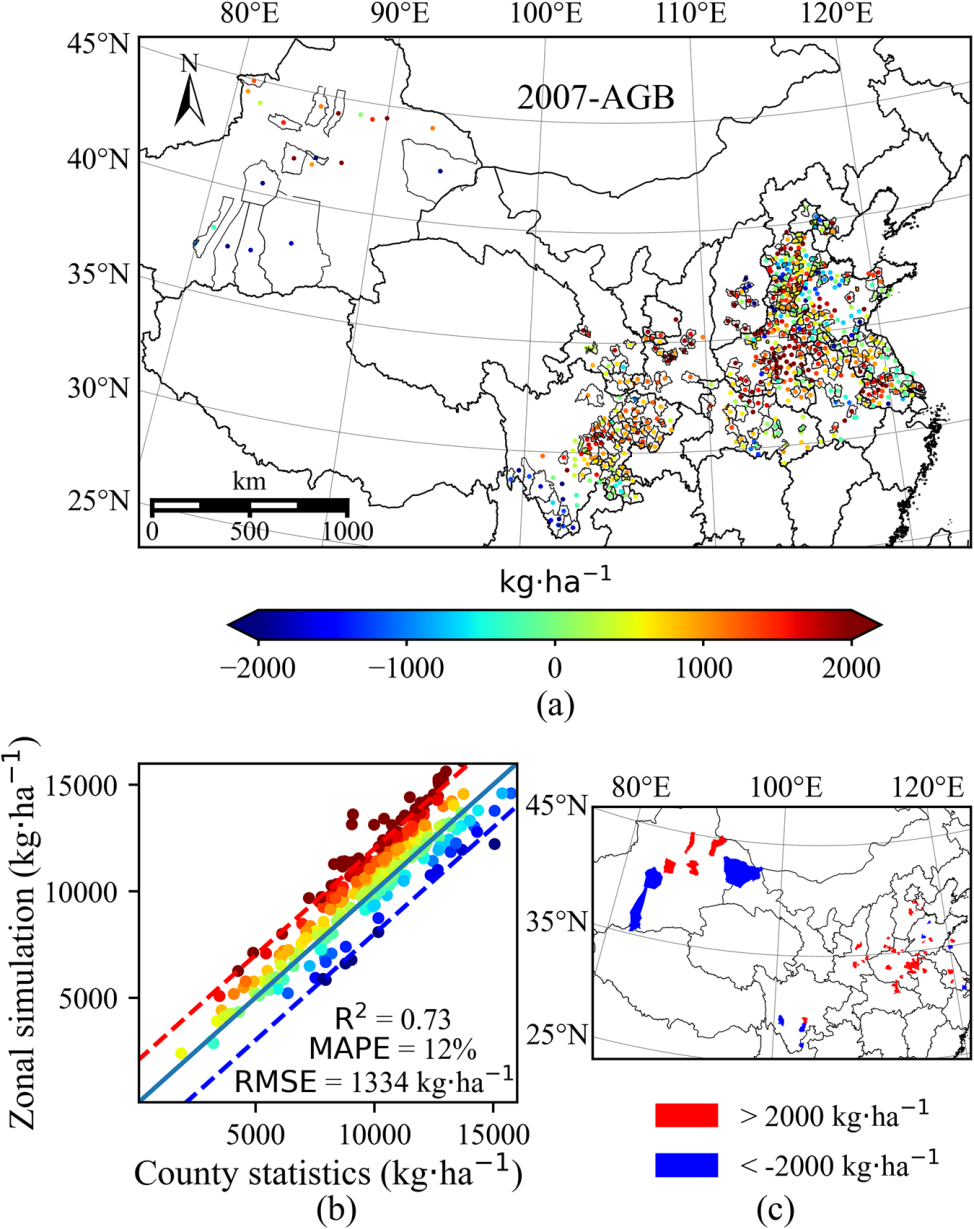
Comparison of county-level simulated and statistical AGB for 2007. (**a**) map of the error distribution. (**b**) R^2^, MAPE, and RMSE between simulated and statistical AGB. (**c**) map of the spatial distribution of counties with errors greater than 2000 kg·ha^−1^ or less than −2000 kg·ha^−1^.

The dataset has a reliable accuracy compared to county-level statistics, the ranges of R^2^ and MAPE are 0.73~0.89 and 8%~12%, respectively (Fig. 7). The total number of used counties for calibration is 348 of 733 counties. The remaining ones, in which the county statistical value and the corresponding calculated statistical value are available, will be used for validation (Fig. 8a). Figure 8 compares the generated dataset with statistics at the county-level scale. The error distribution between simulated and statistical county-level AGB is presented in Fig. 8a, the color represents the difference value between county-level mean simulated and statistical AGB. The data with a range of counties bordered by black lines indicates that the data is used for calibration. Figure 8b shows the comparison of the remaining county statistical data after calibration with the corresponding mean simulated value. The solid line in the middle represents the 1:1 line, and the red and blue dashed lines represent the error equal to 2000 kg·ha^−1^ and −2000 kg·ha^−1^. The added value of the scatterplot shares the same color bar with Fig. 8a. To more clearly show the spatial distribution of counties with large errors, the difference value between county-level mean simulated and statistical AGB greater than 2000 kg·ha^−1^ or less than −2000 kg·ha^−1^ is shown in Fig. 8c. The results for different years are similar, so only the figure for 2007 is shown here, and the comparison for 2008–2015 can be seen in Figures S1–S8. In general, the counties with large errors are mainly divided into two cases. The first is the area with sparse winter wheat planting density, e.g., Xinjiang province, the main reason for the error may come from the influence of the mixed pixels of remote sensing. The second is the counties near the administrative boundary where the statistical data used for the calibration is more prone to large variation in these areas, the error tends to be larger when the calibration data and validation data come from different provinces.

### Uncertainty and limitation

The uncertainty of this data mainly comes from three aspects: (1) the representative error of zonal parameterization caused by the uneven distribution of the stations with observations, (2) the error of the winter wheat classification data, and (3) the mismatch between the 8-day MODIS LAI and the crop growth model simulated LAI on phenological characteristics.

Especially, although the ultimate goal of almost all crop growth models in estimating crop yield (sometimes equivalent to AGB for some crops), simulating AGB to yield is often a less mechanism-based process, usually relying on a set of partitioning factors of the different organs for daily increased biomass or a harvest index at maturity. Therefore, estimating AGB is often more accurate for crop growth models than crop yield and process variables, including LAI^11,12^. We simulated AGB with the WOFOST model starting at the emergence date, and the parameter TDWI determines the value of the start day. The calibration of this parameter remains uncertain due to the lack of observations in the early growth period of winter wheat which is more closely related to TDWI. Therefore, when the AGB is small (in the early stage of winter wheat growth), our data still has a large uncertainty, and the error even exceeds 50%, as shown in Fig. 6.

One of the main factors limiting the further improvement of the accuracy of this dataset is the lack of sufficiently diverse ground observation data for model calibration. We combined statistics and site observation data to calibrate the crop model zonally. Still, some sparse observation data can only get a mathematically optimized parameter calibration result rather than a concurrent one.

## Supplementary information

Supporting Information for A dataset of winter wheat aboveground biomass in China during 2007-2015 based on data assimilation

## Data Availability

Python scripts that implement model calibration, data assimilation, dataset generation, and mapping are available (https://github.com/paperoses/CHN_Winter_Wheat_AGB). Further questions can be directed towards Hai Huang (haihuang@cau.edu.cn).
