# Supplementary material for: A dataset of winter wheat aboveground biomass in China during 2007–2015 based on data assimilation
Source: Sci Data. 2022 May 11;9:200. doi: 10.1038/s41597-022-01305-6 (PMC9095600; doi:10.1038/s41597-022-01305-6)
Supplement: Supplementary file 1 — Supporting Information for A dataset of winter wheat aboveground biomass in China during 2007-2015 based on data assimilation [file 41597_2022_1305_MOESM1_ESM.pdf]

SCIENTIFIC DATA

Supporting Information for

A dataset of winter wheat aboveground biomass in China during 2007-2015 based on data assimilation

Hai Huang[1], Jianxi Huang[1, 2*], Xuecao Li[1, 2,] Wen Zhuo[3], Yantong Wu[4], Quandi Niu[1], Wei Su[1, 2], Wenping Yuan[5]

[1] College of Land Science and Technology, China Agricultural University, Beijing 100083, China
[2] Key Laboratory of Remote Sensing for Agri-Hazards, Ministry of Agriculture and Rural Affairs, Beijing 100083, China
[3] State Key Laboratory of Sever Weather, Chinese Academy of Meteorological Sciences, Beijing, 100081, China
[4] School of Resources and Environment, University of Electronic Science and Technology of China, Chengdu, 611731, China
[5] School of Atmospheric Sciences, Sun Yat-sen University, Guangzhou 510245, Guangdong, China

Table of Contents

Figure S1. Comparison of county-level simulated and statistical AGB for 2008. 3

Figure S2. Comparison of county-level simulated and statistical AGB for 2009. 4

Figure S3. Comparison of county-level simulated and statistical AGB for 2010. 5

Figure S4. Comparison of county-level simulated and statistical AGB for 2011. 6

Figure S5. Comparison of county-level simulated and statistical AGB for 2012. 7

Figure S6. Comparison of county-level simulated and statistical AGB for 2013. 8

Figure S7. Comparison of county-level simulated and statistical AGB for 2014. 9

Figure S8. Comparison of county-level simulated and statistical AGB for 2015. 10

Appendix S1: Quality control of the in-situ measurements at agricultural meteorological stations 11

Table S1. The formulas of conversion from China Meteorological Forcing Dataset to WOFOST model meteorological drive. 12

Table S2. Crop parameters to be calibrated for WOFOST model 13

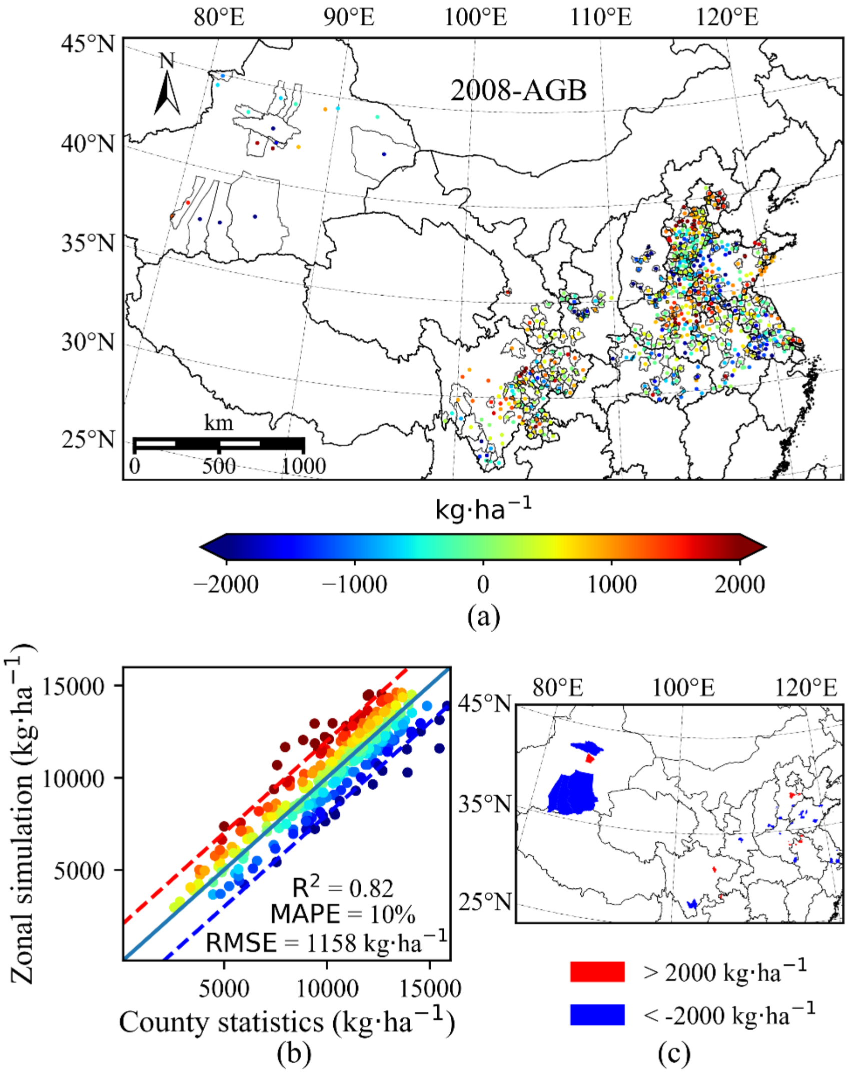

Figure S1. Comparison of county-level simulated and statistical AGB for 2008. (a) map of the error distribution. (b) R2, MAPE, and RMSE between simulated and statistical AGB. (c) map of the spatial distribution of counties with errors greater than 2000 kg·ha^{-1} or less than -2000 kg·ha^{-1}.

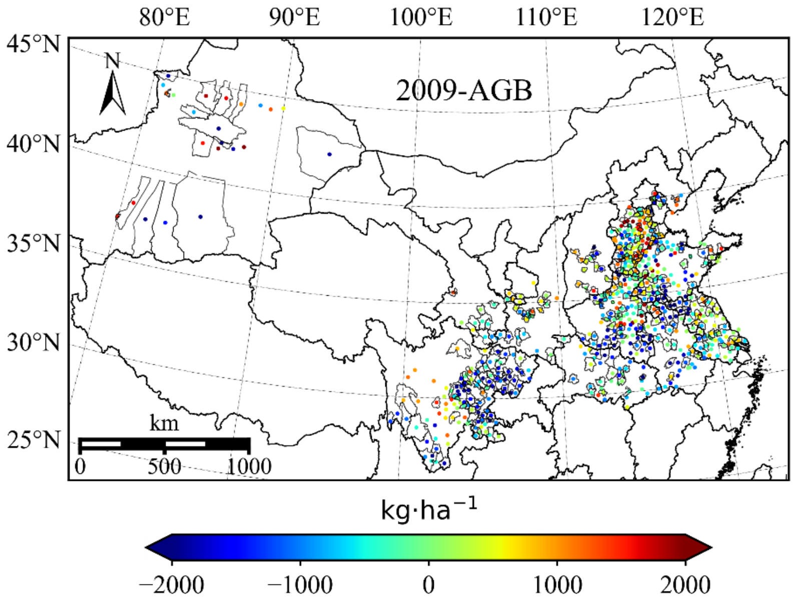

(a)

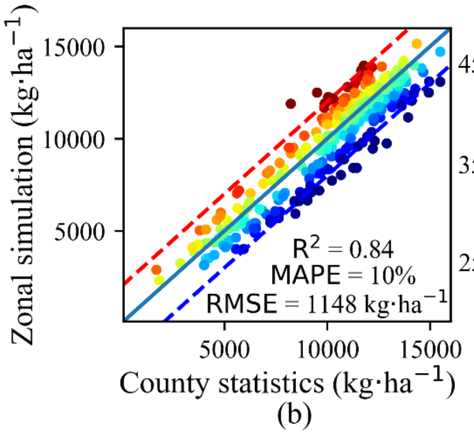

(b)

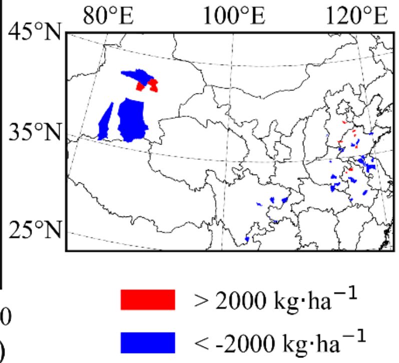

(c)

Figure S2. Comparison of county-level simulated and statistical AGB for 2009. (a) map of the error distribution. (b) R2, MAPE, and RMSE between simulated and statistical AGB. (c) map of the spatial distribution of counties with errors greater than 2000 kg·ha^{-1} or less than -2000 kg·ha^{-1}.

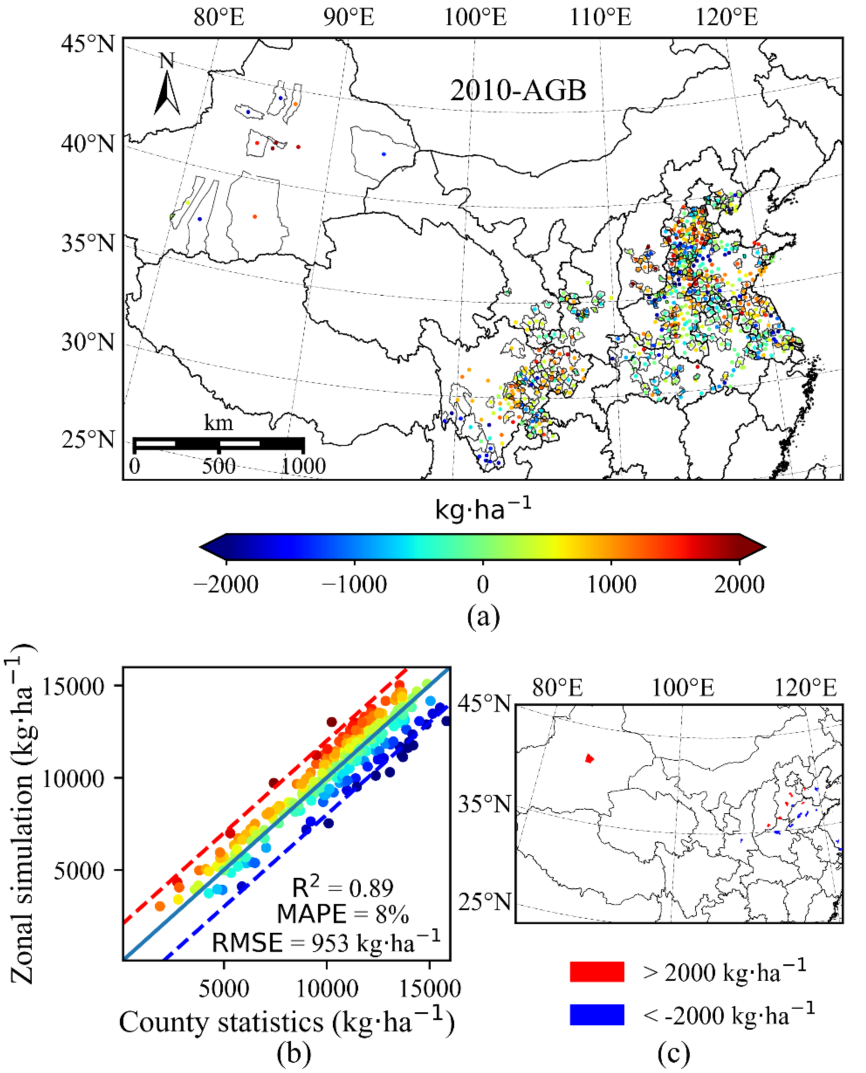

Figure S3. Comparison of county-level simulated and statistical AGB for 2010. (a) map of the error distribution. (b) R2, MAPE, and RMSE between simulated and statistical AGB. (c) map of the spatial distribution of counties with errors greater than 2000 kg·ha^{-1} or less than -2000 kg·ha^{-1}.

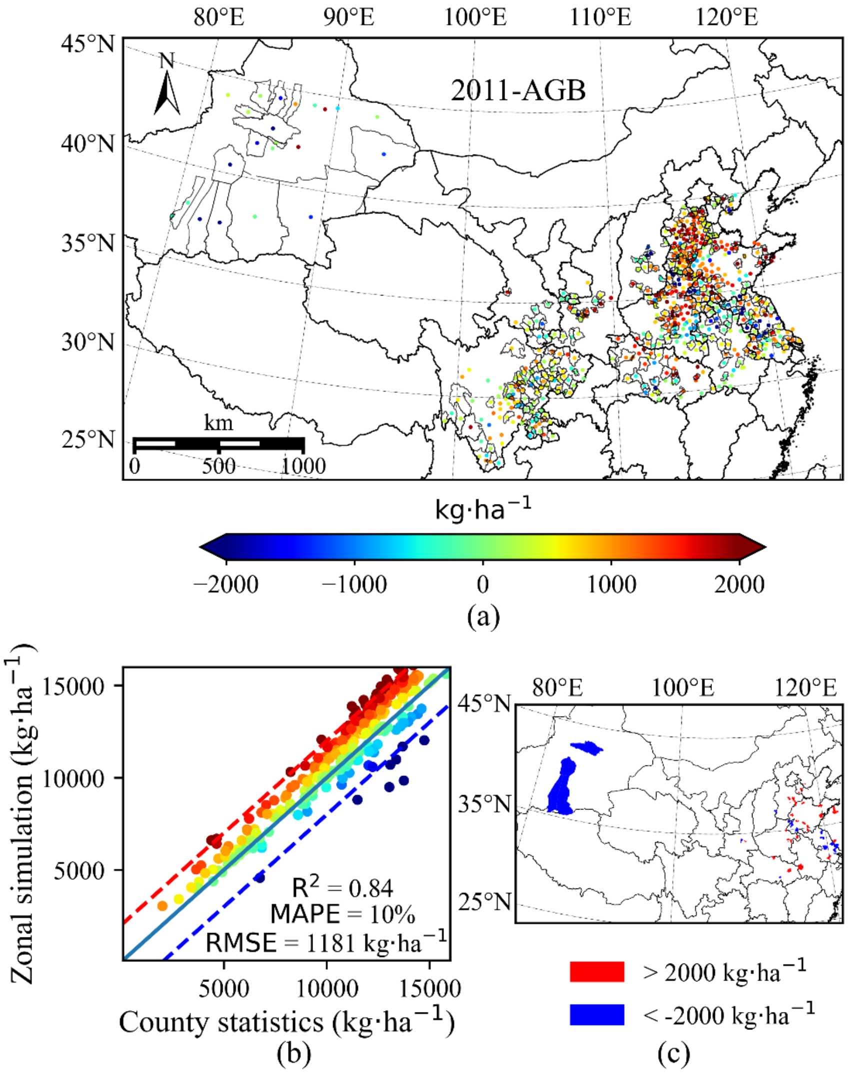

Figure S4. Comparison of county-level simulated and statistical AGB for 2011. (a) map of the error distribution. (b) R2, MAPE, and RMSE between simulated and statistical AGB. (c) map of the spatial distribution of counties with errors greater than 2000 kg·ha^{-1} or less than -2000 kg·ha^{-1}.

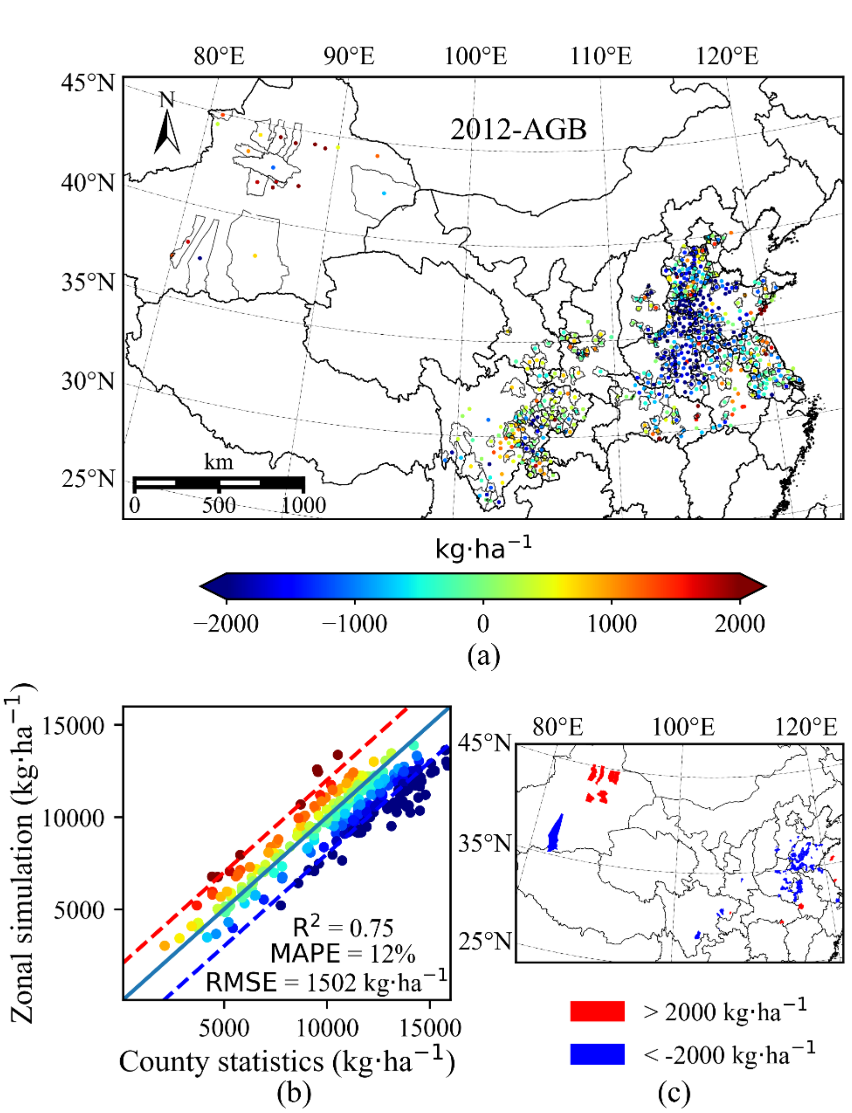

Figure S5. Comparison of county-level simulated and statistical AGB for 2012. (a) map of the error distribution. (b) R2, MAPE, and RMSE between simulated and statistical AGB. (c) map of the spatial distribution of counties with errors greater than 2000 kg·ha^{-1} or less than -2000 kg·ha^{-1}.

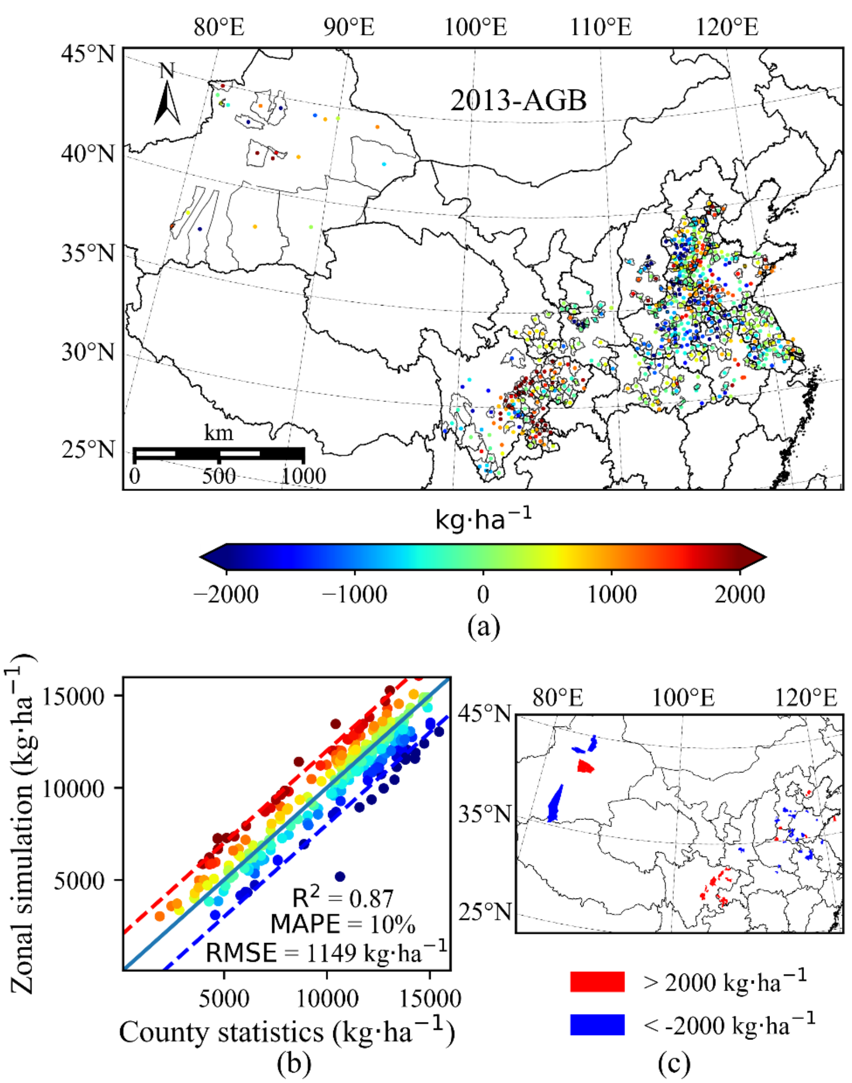

Figure S6. Comparison of county-level simulated and statistical AGB for 2013. (a) map of the error distribution. (b) R2, MAPE, and RMSE between simulated and statistical AGB. (c) map of the spatial distribution of counties with errors greater than 2000 kg·ha^{-1} or less than -2000 kg·ha^{-1}.

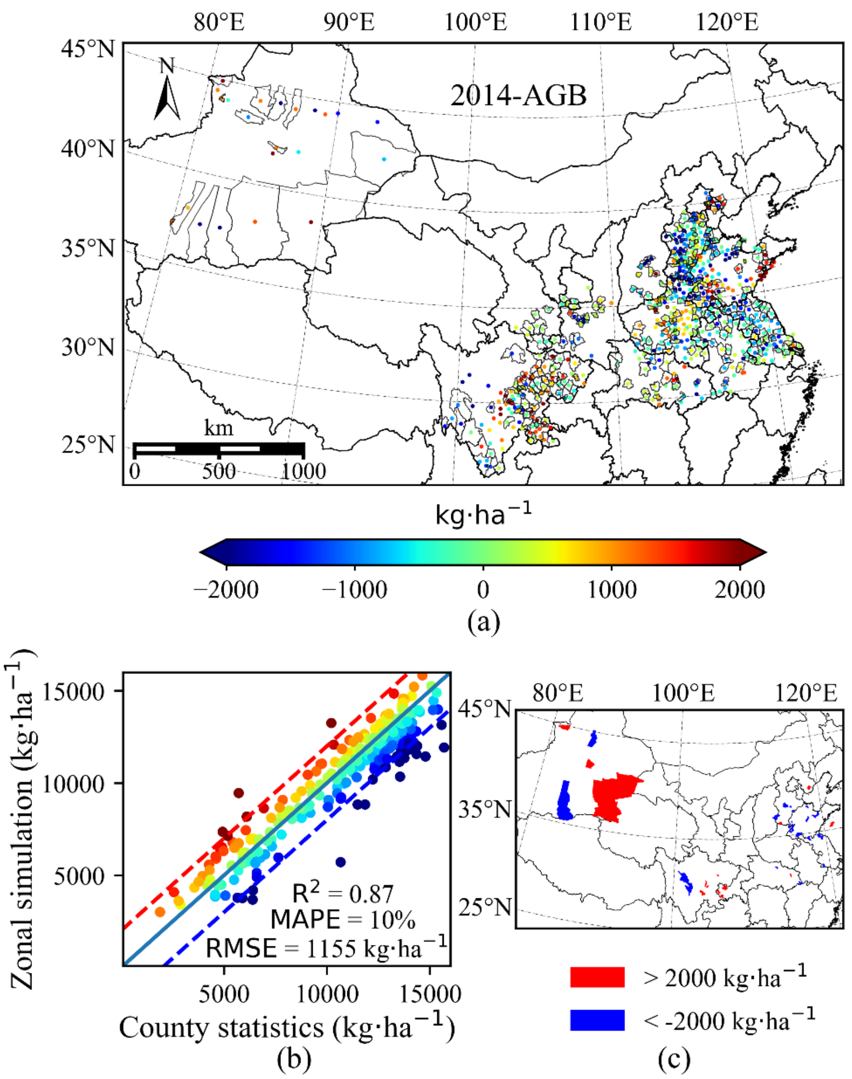

Figure S7. Comparison of county-level simulated and statistical AGB for 2014. (a) map of the error distribution. (b) R2, MAPE, and RMSE between simulated and statistical AGB. (c) map of the spatial distribution of counties with errors greater than 2000 kg·ha^{-1} or less than -2000 kg·ha^{-1}.

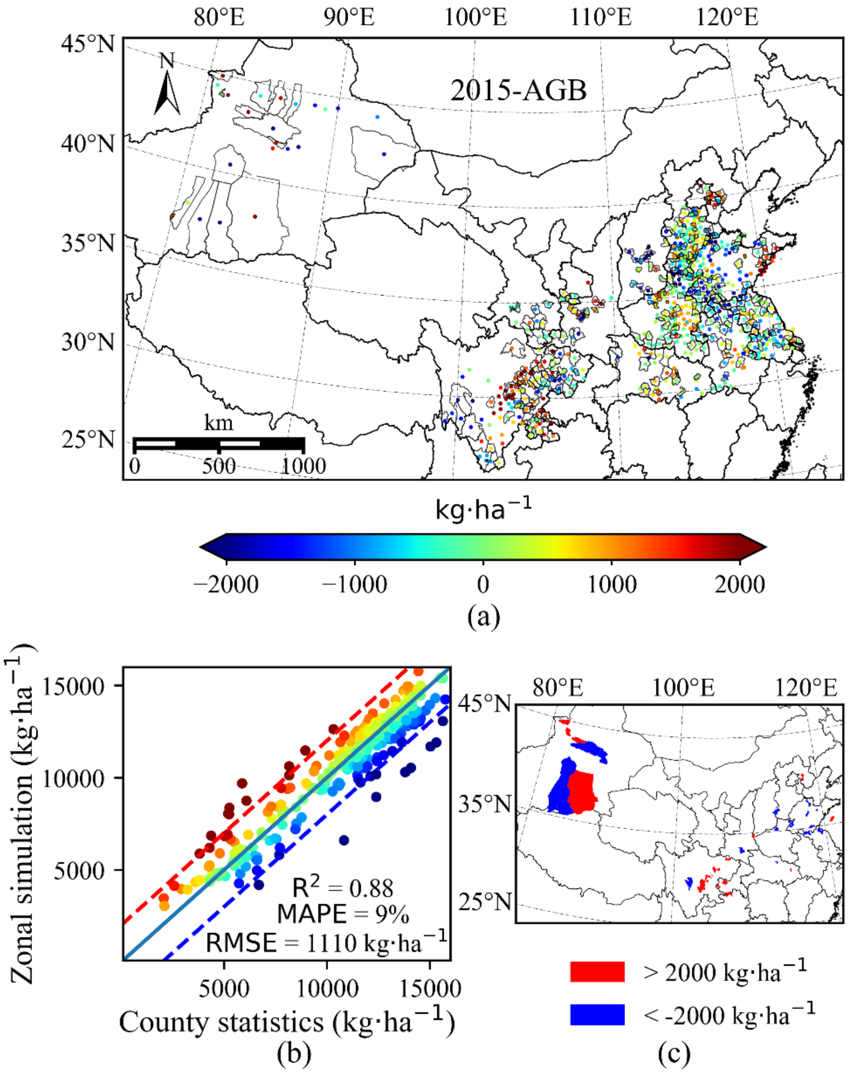

Figure S8. Comparison of county-level simulated and statistical AGB for 2015. (a) map of the error distribution. (b) R2, MAPE, and RMSE between simulated and statistical AGB. (c) map of the spatial distribution of counties with errors greater than 2000 kg·ha^{-1} or less than -2000 kg·ha^{-1}.

Appendix S1: Quality control of the in-situ measurements at agricultural meteorological stations

It is especially noted that the time-series aboveground biomass per unit area ($kg\cdot ha^{-1}$ in this study) at agricultural meteorological stations is estimated as the product of the average plant weight and density, and the calculation formula is as below:

$$P^{O} = \frac{M_{sample}^{O}}{N_{sample}} \times N_{unit\ area}$$

Where P is the biomass production per unit area, the superscript O indicates different wheat organs (leaf, stem, and grain), N_{sample} is the total number of plants sampled, M_{sample}^{O} is the total dry weight of the specific organ biomass sampled, $N_{unit\ area}$ is the total number of plants per unit area.

We carried out quality control inspections and processing on the biomass data to reduce the impact of sampling errors based on the calculated harvest index (HI):

$$HI = \frac{Y}{AGB_{mature}}$$

Where Y is the field measured grain yield after harvest, AGB_{mature} equals to the sum of P^{leaf} , P^{stem} and P^{grain} at maturity stage. If the calculated HI is abnormally small (< 0.3) or abnormally large (> 0.7), this situation is usually caused by the unrepresentative $N_{unit\ area}$. We then recalculated AGB as AGB_{adjust}:

$$AGB_{adjust} = \frac{AGB_{mature}}{P^{grain}} \times Y$$

Besides, within-season AGB cannot decrease throughout the growth period. But some data have an abnormal reduction. This is due to the inconsistency of wheat growth situation in the sampling locations before and after, and on the other hand, sampling error caused by leaf wilting and shedding in the late growth period. Therefore, when a certain AGB data is unusually less than 90% of the maximum value of the previous data, we regard it as untrusted data d discard it.

Table S1. The formulas of conversion from China Meteorological Forcing Dataset to WOFOST model meteorological drive.

Meteorological element	Unit	conversion from China Meteorological Forcing Dataset
radiation	$kJ \cdot m^{-2} \cdot d^{-1}$	Srad×24×3.6
minimum temperature	℃	min(Temp)-273.15
maximum temperature	℃	max(Temp)-273.15
water vapor pressure	kPa	Shum×Pres/(0.622+Shum×0.378)/1000
average wind speed	$m \cdot s^{-1}$	V_{wind}
precipitation	$mm \cdot d^{-1}$	Prec×24

Note: Srad, Temp, Shum, Pres, V_{wind}, and Prec represent daily downward shortwave radiation ($W \cdot m^{-2}$), 3-hour near-surface air temperature within one day (K), daily near-surface total humidity ($kg \cdot kg^{-1}$), daily near-surface air pressure (Pa), daily wind speed ($m \cdot s^{-1}$) and daily ground precipitation rate ($mm \cdot hr^{-1}$), respectively.

Table S2. Crop parameters to be calibrated for WOFOST model

Parameter	Description	Units
TSUM1	the thermal time from emergence to anthesis	℃·d^{-1}
TSUM2	the thermal time from anthesis to maturity	℃·d^{-1}
DTSMTB	the daily increase in temperature sum as a function of temperature	℃, -
SLATB	specific leaf area as a function of the development stage	-, ha·kg^{-1}
SPAN	the life span of leaves growing at an average temperature of 35 ℃	d
AMAXTB	maximum CO^2 assimilation rate as a function of the development stage of the crop	-, kg·ha^{-1}·h^{-1}
TMPFTB	a correction factor of maximum leaf CO^2 assimilation rate as a function of average daily temperatures	℃, -
TMNFTB	a correction factor of daily gross CO^2 assimilation rate as a function of minimum temperature	℃, -
CVO	efficiency conversion of assimilates into storage organ dry matter	kg·kg^{-1}
FSTB	fraction of total dry matter to stems as a function development stage	-, -
FOTB	fraction of total dry matter to storage organs as a function of the development stage	-, -
TDWI	initial total crop dry weight	kg·ha^{-1}

Note: All parameters ending in "TB" are a piecewise linear function in the form of a one-dimensional array with paired data. The uneven places in the array represent the X-values, whereas the even places of the array represent the Y-values. In this study, DTSMTB was optimized by changing the base temperature of effective accumulated temperature, which default is 0. SLATB and AMAXTB were optimized by being simplified to a constant function. TMPFTB was optimized by changing a value in the array, representing the maximum optimal average temperature value for the maximum leaf CO^2 assimilation rate. TMPFTB was optimized by identically shifting the minimum temperature when the correction factor is 0 and 1. FSTB and FOTB were optimized by adjusting the same value, the corresponding development stage when the total dry matter is first allocated to storage organs. The default crop parameter for wheat was sourced from https://github.com/ajwdewit/WOFOST_crop_parameters.
